# Internet Use and Quality of Life: The Multiple Mediating Effects of Risk Perception and Internet Addiction

**DOI:** 10.3390/ijerph19031795

**Published:** 2022-02-04

**Authors:** Bo Qian, Mengmeng Huang, Mengyi Xu, Yuxiang Hong

**Affiliations:** 1 School of Management, Hangzhou Dianzi University, Hangzhou 310018, China; qianbo@hdu.edu.cn (B.Q.); 18031208@hdu.edu.cn (M.H.); 2 Cranfield School of Management, Cranfield University, College Road Cranfield, Bedfordshire MK43 0AL, UK; mengyi.xu@cranfield.ac.uk

**Keywords:** quality of life, internet use, risk perception, internet addiction, multiple mediating effects

## Abstract

The impact of internet use on quality of life (QoL) has become an increasing focus of academic research. This paper aims to explore the internal influencing mechanisms of internet use (i.e., leisure-oriented internet use (LIU); work-oriented internet use (WIU)) on QoL, with a focus on the multiple mediating effects of risk perception and internet addiction. We constructed a theoretical framework from a psychological perspective and tested the hypotheses using hierarchical regression analysis with a sample of 1535 participants. The results showed that: (1) LIU had a positive effect on QoL, while WIU did not have a significant impact on QoL; (2) both risk perception and internet addiction had a negative influence on QoL; (3) risk perception positively impacted internet addiction; (4) risk perception and internet addiction had multiple mediating effects on the relationship between internet use and QoL.

## 1. Introduction

Health researchers, clinicians, and policymakers currently regard quality of life (QoL) as an indicator when quantifying the health problems of human society [1,2]. Although there is no unified definition of QoL, most definitions relate to a global perception of physical function, mental health, and happiness [3,4,5]. Previous studies found that many factors lead to an improvement or deterioration in QoL, such as personality, work environment, leisure, and social capital [6,7]. With technological advancement and digital transformation, the use of the internet has been ubiquitously dominated in our work and life in various ways. Most of the existing literature has studied the classification of internet use. For example, literature [8] presented three functions of internet use: social services, information services, and leisure services. Literature [9] proposed four types of internet services: work, entertainment, social interaction, and family use. Recently, some studies had pay attention to the relationship between internet use and QoL as well as the relationship between internet addiction and QoL [10,11]. However, there is limited understanding of the internal influencing mechanism that different forms of internet use impact on people’s QoL, and how this applies to the development of sustainable and healthy internet use practices that can help frame more valid institutional interventions benefitting the contemporary netizen and the digital human society.

Our study aims to explore the effects of different types of internet use on QoL, especially the internal influencing mechanisms resulted from risk perception and internet addiction. Based on previous studies, we divided and examined the following two types of internet use for test: (1) work-oriented internet use (WIU), which refers to using the internet for work and study purposes; and (2) leisure-oriented internet use (LIU), which refers to using the internet for leisure purposes (e.g., chatting online, playing games). Meanwhile, we also tested the mediating effects of risk perception and internet addiction on the relationship between internet use and QoL. Risk perception is an individual’s psychological reaction based on their judgement of the scope and degree of specific risks and depending on their knowledge system, which can be considered a negative predictor of QoL [12]. Based on the social amplification of risk framework (SARF) [13], internet use can be predicted to have a significant amplification effect on an individual’s risk perception, which will impact QoL. Furthermore, some empirical studies found a significant relationship between internet use and internet addiction. Internet addiction refers to a state, whereby an individual has a strong dependence on the internet and has lost control of their internet use [14]. The harmful effects of internet addiction on health or QoL have been verified in previous studies [15,16].

This study has the following contributions. First, by dividing internet use into two types, we intend to offer a new and subtle angle to analyse the influences of internet use on QoL in the internet era and risk society. The positive effect that LIU has on QoL was verified in this study. Moreover, differences between the two types of internet use were also found, such as that LIU has a positive effect on risk perception, while the effect of WIU impacting risk perception is negative; LIU impacts internet addiction more than WIU. Second, this research is destined to expand the positive psychology and well-being literature by highlighting the complexity of internal influencing mechanisms of internet use on QoL. In contrast, extant research into psychological mechanisms is less conclusive despite the ongoing efforts to carry out discussions on internet use and well-being [17]. A serial multiple mediating effect between LIU and QoL was verified with the path: LIU → + risk perception → +internet addiction → −QoL. Third, this is an interdisciplinary study that combines theories of psychology and communication, to comprehensively explain the influencing mechanisms of internet use on QoL.

## 2. Theoretical Framework and Hypotheses Development

### 2.1. Internet Use and QoL

Internet use and QoL is argued to be correlated. Uses and gratification theory indicates that individuals use media proactively to meet their psychological and social needs [18], thus spending time on specific media to fulfil their expected gratifications [19]. According to self-determination theory, the satisfaction of individuals’ psychological needs (e.g., autonomy, competence, and relatedness) will positively predict their life satisfaction, happiness, and well-being [20]. Therefore, in terms of internet use, when individuals use the internet for leisure or work, their needs can be satisfied, which will, in turn, generate positive long-term psychological outcomes, such as QoL [21,22]. 

Since different purposes of internet use satisfy different personal needs, in this study, we analyzed the effects of two types of internet use on QoL including LIU and WIU through different influencing mechanisms. Regarding LIU, on the one hand, leisure reduces stress and contributes to health and well-being [23,24,25]. The internet expands the forms and content of entertainment and magnifies the utility of leisure. However, the internet also possesses features, such as decentralization, rapid cross-regional dissemination, and low cost of interpersonal communication. Leisure-oriented internet use enables people to meet like-minded fellows, thereby generating social support, social identity, and a sense of belonging. Thus, it is beneficial to the formation of intimacy and the establishment of interpersonal relatedness [26], which satisfies the need for relationships and improves QoL [20]. Regarding WIU, using the internet helps individuals communicate their problems and complete tasks more efficiently compared to non-virtual approaches, thereby satisfying the need for competence and generating a high level of QoL [20]. Therefore, the hypotheses were proposed as follows:

**Hypothesis** **1** **(H1).** *Leisure-oriented internet use has a positive effect on QoL.*

**Hypothesis** **2** **(H2).** *Work-oriented internet use has a positive effect on QoL.*

### 2.2. Risk Perception

While economic development and technological advancement greatly improve people’s living standards around the globe, the vulnerability of society has increased with various risks soaring, such as environmental risks, health risks, and epidemic outbreaks. Risk became a basic element of modern social and political agendas since 1990 [27] and remains the priority now and future [28]. Facing risks to the external environment, the individual’s sense of crisis and insecurity has the potential to cause anxiety. This anxiety stems from the individual’s uncertainty and uncontrollability of the unknown, which breeds hopelessness and reduces the QoL of the individual. The negative effect of risk perception on QoL has been verified in different scenarios, such as the risk perceptions of crime [29], political, economic and communitarian security [30], health [31], natural disaster [12,32]. Therefore, the following hypothesis was proposed:

**Hypothesis** **3** **(H3).** *Risk perception has a negative effect on QoL.*

According to SARF, risk is socially constructed by information process, social and institutional environments, and individual responses [13]. Nowadays, the internet is usually considered to be an important “social station” for the social amplification of risk by providing the indirect experience of the disasters and risks [33,34].

Compared with a traditional industrial society, the current digital society can cause a potential increase in individuals’ risk perception to a certain extent. This is because people’s perception of the objective world is mainly constructed based on the information obtained, through which the internet is argued to be the main source. Internet information has the features self-agency, interactivity, multi-form, timeliness, and a high level of accessibility and dissemination [35,36,37]. The use of the internet can play a positive role in the formation of QoL in terms of the satisfaction of needs. However, it also exposes individuals to the broader range of the world where embeded with dark aspects such as infectious diseases, natural disasters, social conflicts, and wars, which increases risk perception and weakens the positive direct effects on QoL. Internet information can be used to report, explain, attribute, and construct risks and emergencies, from where individuals are likely to cognize the real world and form stronger risk perceptions. Moreover, the internet can disseminate misinformation rapidly because of instant and various media channels and limited time for fact-checking; individuals who lack adequate cognitive abilities can be easily misled, which amplify the risk perception [38]. Therefore, internet use can be an important factor affecting personal risk perception. 

Different types of internet use relying on various information sources can produce different information dissemination and have risk-amplification effects. The information sources used in WIU are biased toward official media and documentation. They are often more formal, more accurate, and less erroneous. By contrast, while the information used in LIU is thoroughly processed and packaged many times, the authenticity is relatively low. Due to the nature of the internet, individuals will, inevitably and to varying degrees, be exposed to erroneous or/and exaggerated risk information when using the internet for leisure. The more that individuals use the internet to access risk information, the more likely they are aware of negative aspects of society, thus forming stronger risk perceptions. Therefore, the following hypotheses were proposed:

**Hypothesis** **4** **(H4).** *Leisure-oriented internet use has a positive effect on risk perception*.

**Hypothesis** **5** **(H5).** *Work-oriented internet use has a positive effect on risk perception.*

**Hypothesis** **6** **(H6).** *Leisure-oriented internet use impacts risk perception more than work-oriented internet use.*

### 2.3. Internet Addiction

The internet can provide important support for entertaining (e.g., playing online games), travel (e.g., searching for the best route before going out), therapy (e.g., searching for medication methods online when sick), social connectedness (e.g., making friends on social media), and work (e.g., online meetings, sending e-mails). However, excessive internet use might result in physical or psychological dependence and may even become addictive [39]. A meta-analysis of one hundred studies on internet addiction and found that time spent in internet use positively predicted internet addiction [40]. Moreover, different types of internet use might also have different impacts on internet addiction. This is evidenced in previous studies that people diagnosed as internet addicts invested more time in LIU (e.g., online chatting, internet games) and less time in WIU (e.g., using search engines) than non-addicts [41,42,43]. Therefore, we hypothesized that:

**Hypothesis** **7** **(H7).** *Leisure-oriented internet use impacts internet addiction positively.*

**Hypothesis** **8** **(H8).** *Work-oriented internet use impacts internet addiction positively.*

**Hypothesis** **9** **(H9).** *Leisure-oriented internet use impacts internet addiction more than work-oriented internet use.*

Internet addiction has similar symptoms to alcohol, gambling, or drug addiction, which are usually associated with unhealthy lifestyles, such as failure in school, poor performance at work, and family discord [43]. Such addiction can result in harmful reactions to both an individual’s physical and mental health, such as a higher level of depression severity, anxiety, stress, and poor sleep quality, all of which leads to poor QoL [44,45,46,47]. Moreover, keeping internet use at a normal level can be beneficial; as excessive reliance on the internet makes individuals disconnected from the real world, their emotional support and social bonds can be significantly weakened, contributing to poor QoL [48]. Hypothesis 10 was thus proposed:

**Hypothesis** **10** **(H10).** *Internet addiction has a negative effect on QoL.*

People who perceive increasing risk information tend to compensate from other channels to maintain balanced psychological wellbeing. Compensation as a type of psychological adaptation mechanism refers to an individual’s efforts to make up for a loss, and gain a sense of superiority when frustrated in pursuit of a specific goal or abased by some defect [49]. Individuals seeking to compensate and reducing feelings of powerlessness is an aversive psychological state [50]. When individuals perceive risks in the world around them and are unable to avoid them (e.g., natural/social/cyber disasters), they tend to suffer a sense of powerlessness or frustration, and autonomously seek compensation in certain ways. In the digital society, internet use is considered an important compensatory behavior, due to its features of anonymity, deindividuation, and low pressure [51]. The compensatory internet use theory argues that people will compensate through internet use behaviors if they are exposed to too much risk information in real life [14]. In other words, individuals might need to use the internet to psychologically trade-off the life issues they experienced and the cruelty of the real world around them, which increases their dependence and addiction to the internet [52]. Thus, we hypothesized that:

**Hypothesis** **11** **(H11).** *Risk perception has a positive effect on internet addiction.*

### 2.4. The Multiple Mediating Roles of Risk Perception and Internet Addiction

Based on the previous analysis, multiple mediation effects might exist among LIU/WIU, risk perception, internet addiction, and QoL, leading to three indirect paths [53], by which LIU/WIU affected QoL through at least one mediator (RP or IA): (1) LIU/WIU → risk perception → QoL; (2) LIU/WIU → internet addiction → QoL; (3) LIU/WIU → risk perception → internet addiction → QoL. Therefore, the following hypotheses were proposed:

**Hypothesis** **12** **(H12).** *Risk perception and internet addiction have serial multiple mediating effects between leisure-oriented internet use and QoL.*

**Hypothesis** **13** **(H13).** *Risk perception and internet addiction have serial multiple mediating effects between work-oriented internet use and QoL.*

**Figure 1:**
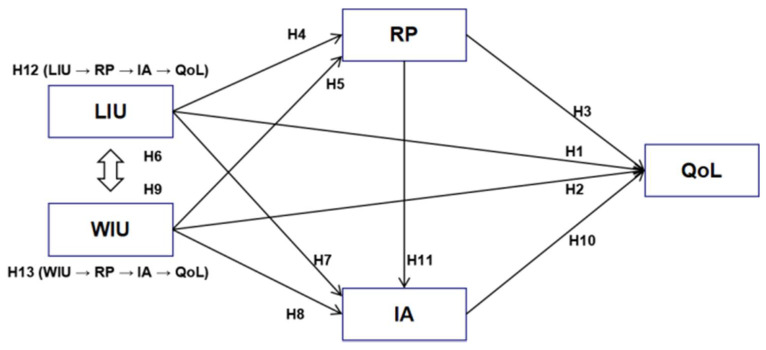
Theoretical framework.

The theoretical framework of this study is provided in Figure 1.

## 3. Method

### 3.1. Study Participants

The data used in this study were from the 2019 “Taiwan Social Change Survey” (TSCS) with the theme of “Technology and Risk Society”, hosted by the Academia Sinica’s Institute of Sociology. A sample size of 4054 was originally decided based on stratified multi-stage probability proportional to size sampling with the household registration data as the sample box. A total of 1933 adult participants finished the survey by door-to-door interviews. The effective response rate was 48%. After removing missing and invalid data, 1535 subjects were retained, including 830 (54.1%) men and 705 (45.9%) women. The average age of the participants was 42 years (SD = 14.852). The socio-demographic information of the participants is presented in the first column of Table 1.

### 3.2. Measures

#### 3.2.1. Quality of Life

QoL was measured based on the studies of [12] and [54], including three items: (1) “How happy or unhappy are you overall? (Not happy at all to very happy)”; (2) “How satisfied are you with your life nowadays? (Not satisfied at all to very satisfied)”; (3) “How would you say your physical health has been for the past two weeks? (Poor to very good)”. All the items had a five-point Likert scale ranging from 1 to 5.

#### 3.2.2. Internet Use

Leisure-oriented internet use was measured by one item: “In the past year, how often have you used social networking sites or communication software (such as Facebook, blogs, YouTube, Line, Skype, WeChat, etc.) to chat, connect, play games, share videos, etc.?”. WIU was also measured by one item: “In the last year, how often have you bought and sold things, done things or work through the internet? (for example: browse or inquire information, send and receive e-mails, pay taxes online, buy and sell stocks, book accommodation, buy air tickets, and so on”. All the items had a six-point Likert scale ranging from 1 (never) to 6 (several times a day).

#### 3.2.3. Risk Perception

Similar to previous studies [55,56,57,58], risk perception was measured using four items: (1) “Do you worry about yourself or your family being affected by a hurricane or flooding”; (2) “Do you worry about yourself or a family member being hurt in an earthquake”; (3) “Do you worry about your own or your family’s work environment resulting in injury or illness”; (4) “Do you usually worry about yourself or your family being involved in a traffic accident?”. All the items used a scale ranging from 1 (not at all worried) to 5 (extremely worried).

#### 3.2.4. Internet Addiction

Internet addiction was measured using five items, asking “In the past 12 months, have you met the following criteria: (1) Always wanted to use social networking sites or communication software; (2) Used social networking sites or communications software to forget personal problems; (3) Tried to reduce time spent on social networking sites or communications software, but did not succeed; (4) Feel distressed or restless when it is not possible to use social networking sites or communications software; (5) Overly frequent use of social networking sites or communications software had a negative impact on studies/work”. All the items used a scale ranging from 1 (not met at all) to 6 (totally met).

### 3.3. Data Analysis

We tested the adequacy of measurements using confirmatory factor analysis (CFA), tested the common method bias (CMB) using Harman’s (1967) single-factor test [59], tested the difference in LIU, WIU, risk perception, internet addiction, and QoL by gender, age, and education using one-way analysis of variance (ANOVA), and tested the hypotheses with multiple regression analysis. Then, we used PROCESS (Model 6) to test the serial multiple mediating effects of risk perception and internet addiction, which can be translated into three equations:

RP = β_01_ + β_1_LIU/WIU + ε_1_ (1)

IA = β_02_ + β_2_RP + β_5_LIU/WIU + ε_2_ (2)

QoL = β_03_ + β_4_LIU/WIU + β_3_IA + β_6_RP + ε_3_ (3)

SPSS ver. 26 (IBM, Armonk, NY, USA) was used to analyze the data. 

## 4. Results

### 4.1. Preliminary Analysis 

Prior to data analysis, internal consistency reliability analysis and CFA were conducted to test the quality of the scales and sample. As common measure of reliability, Cronbach’s α coefficient was used to check internal consistency. The results showed that Cronbach’s α of risk perception was 0.83, internet addiction was 0.763, and QoL was 0.711, which presents a high internal consistency. 

We then conducted CFA on risk perception, internet addiction, and QoL. The Kaiser–Meyer–Olkin value was 0.761 (greater than 0.7); Bartlett’s test of sphericity was significant, indicating that the sample size is sufficient and the data can be analyzed by factor analysis. As can be seen from Table 2, the loadings of all items were greater than 0.5, the composite reliability values were greater than 0.7, and the average variance extraction (AVE) values were greater than 0.5. The convergent validity was thus good. Moreover, the square root of AVE for each construct was greater than the correlation coefficient between it and all other constructs, indicating a good discriminant validity [60].

The correlation coefficients among the variables are reported in Table 3. Moreover, as all the measures of the variables were obtained from the same source, which could result in the CMB, our study used Harman’s (1967) single-factor test [59] to test for CMB. The results showed that 21.7% of the variance could be attributed to the largest factor and that four factors could explain 61.6% of the variance, which indicated a low risk of CMB.

Additionally, based on the results of ANOVA, a statistically significant difference between groups of men and women can be found, such as women (mean = 5.41) spending more time on LIU than men (mean = 5.27) and risk perception being higher in women (mean = 3.39) compared to men (mean = 3.11). Regarding age, younger people spent more time on LIU and internet addiction was higher in younger groups. Regarding education, better-educated people spent more time on WIU and had a lower level of risk perception. 

### 4.2. Hypotheses Testing 

As shown in Table 4, we constructed regression models for QoL, risk perception, and internet addiction, respectively. The regression model (Model 1) was constructed for QoL using the control variables, WIU, LIU, risk perception, and internet addiction; the results showed that LIU had a positive effect on QoL (Model 1, β = 0.056, *p* < 0.01). Hypothesis 1 was thus supported and the result indicated that both risk perception (Model 1, β = −0.058, *p* < 0.01) and internet addiction (Model 1, β = −0.067, *p* < 0.01) had negative effects on QoL. Hypotheses 3 and 10 were thus supported. However, the effect of WIU impacting QoL was not significant (Model 1, β = −0.015, *p* > 0.05). Considering risk perception and internet addiction might totally mediate the relationship between WIU and QoL, we conducted the regression based on the model excluding risk perception, internet addiction, and LIU, and the effects of WIU were also not significant. Hypotheses 2 was thus not supported. A possible explanation could be the existence of some mediation mechanisms that go in the opposite direction, which we will discuss in the following part of mediating effect. Then, we constructed a regression model (Model 2) for internet addiction, with the results indicating that LIU (Model 2, β = 0.201, *p* < 0.01), WIU (Model 2, β = 0.072, *p* < 0.01), and risk perception (Model 2, β = 0.097, *p* < 0.01) positively impact on internet addiction. Hypotheses 7, 8, 9, and 11 were thus supported. We also conducted a regression (Model 3) for risk perception, with the results indicating that LIU has a positive effect on risk perception (Model 3, β = 0.084, *p* < 0.01), while the effect of WIU impacting risk perception is negative (Model 3, β = −0.034, *p* < 0.05). Hypotheses 4 and 6 were thus supported, while Hypothesis 5 was not supported.

To test Hypothesis 12, we utilized the PROCESS (Model 6) provided by Hayes (2013) [53]. We estimated 5000 bootstrap samples, in which the independent variable was LIU, the mediator was risk perception and internet addiction, and the dependent variable was QoL. We also included gender, age, and education as covariates in the model. Specifically, the mediation effect was generated through three mediation chains: LIU → risk perception → QoL; (2) LIU → internet addiction → QoL; (3) LIU → risk perception → internet addiction → QoL. As shown in Table 5, the results indicated that risk perception mediated the relationship between LIU and QoL (indirect effect = −0.0039; 95% CI (−0.0083, −0.0008)); internet addiction mediated the relationship between LIU and QoL (indirect effect = −0.0163; 95% CI (−0.0276, −0.0064)); risk perception and internet addiction had a serial mediating effect on the relationship between LIU and QoL (indirect effect = −0.0004; 95% CI (−0.0010, −0.0001)). Therefore, Hypothesis 12 was supported. The same method was conducted to test Hypothesis 13, as shown in Table 6, with the results indicating that internet addiction mediated the relationship between WIU and QoL (indirect effect = −0.0060; 95% CI (−0.0112, −0.0015)); risk perception and internet addiction had a serial mediating effect on the relationship between WIU and QoL (indirect effect = 0.0001; 95% CI (<0.0001, 0.0005)). These mediating effects had opposite directions, which explain why Hypothesis 2 was not supported.

## 5. Discussion

Our findings revealed a more complicated relationship between the two types of internet use and people’s quality of life than most extant literature has noted. Based on the conceptual model developed by our study, most of the hypotheses were supported. First, we found that different types of internet use had different impacts on QoL. The empirical test results indicated that LIU had a positive effect on QoL, while the impact of WIU was not significant. Therefore, compared to WIU, LIU is more advantageous to the raising of QoL. This finding was consistent with some previous studies related to LIU, especially the literature discussing the role of social media [61,62,63]. 

Second, we explored the serial mediating roles of risk perception and internet addiction in the relationship between the two types of internet use and QoL. On the one hand, risk perception is an important predictor of changes in QoL [12]. The internet has become one of the most common and unconscious ways for individuals to receive risk information and make judgments, so the role of risk perception could not be ignored when discussing internet use. We also found that LIU had a positive effect on risk perception, while the effect of WIU was not significant. One possible explanation could be that tools for WIU are usually official that inherently bundled within the organizational discipline and individual compliance, which to some extent filter out extreme or unsupported information for users/employees. Moreover, people spending time on WIU tend to consider the financial stability, which has been proven to increase an individual’s self-sufficiency and decrease their concern about the surroundings, thus decreasing their risk perception [64]. On the other hand, we also considered the influences of internet addiction in the relationship between internet use and QoL. Inconsistent with previous studies [47], we found that internet addiction can negatively impact QoL. We verified the previous literature (e.g., [42]) that both LIU and WIU lead to internet addiction, that is, the more time people spend on LIU, the higher the possibility of internet addiction compared to WIU. The internet has been deeply woven into our daily lives more than ever for both entertainment and work purposes. Social media and short video apps, in particular, have enriched people’s lives in various ways and increased connectivity with diverse communities, making them difficult to live without. In addition, consistent with compensatory internet use theory, the positive effect of risk perception on internet addiction was also verified. In societies at risk, especially in the wake of COVID-19, individuals directly or indirectly experience more physical and psychological disasters, which increase their risk of internet addiction.

### 5.1. Implications for Research

The study contributes to the advancement of psychology and wellbeing literature in the following ways. First, by dividing internet use into two major functional types (LIU/WIU) according to different purposes, we explored the two types of influencing mechanisms of internet use by comparing their effects on QoL through risk perception and internet addiction. Little previous literature paid attention to a comparative study of the specific effects on different types of internet use, and almost all of them simply defined this variable as the sum of all internet usage time or focused on internet use as a whole. Our study is a step forward from this research, indicating that understanding the nature of different internet use is the primary task of explaining their effects on people’s psychology or behaviors. 

Second, our study broadens the research scope by revealing a more complicated process than previously expected on how internet use impacts people’s QoL by considering the role of risk perception and internet addiction. Many previous studies have focused on whether the relationship between internet use and QoL is positive [65] or negative [66]. However, these research results have almost no consensus and do not connect the relationships between various factors. Our study tested and validated the mediating effects of internet addiction and risk perception on the relationship between internet use and QoL. In the internet era and social society, these two factors could not be neglected when discussing internet use or QoL. The findings of the internal influencing mechanisms provide more insight into the impact of internet use.

Third, the findings of this study provide fresh insight into a growing body of psychology and wellbeing literature by integrating theories concerning communication (i.e., compensation internet use) in analysing the effect of internet use on QoL in the digital society. Previous studies on QoL are mainly based on psychological theories to study the factors that affect QoL. Considering the complexity of internet use to QoL through risk perception and internet addiction, we combined theories from both psychology and communication to comprehensively explain the influencing mechanisms of internet use on QoL.

### 5.2. Implications for Practice

Understanding the influence of internet use on QoL can greatly provide implications for practice. QoL is not only the issue of individuals but becomes an important political and societal agenda nationally and internationally [67,68]. Therefore, multiple organisations, including the government, enterprises, communities, social organizations, and individuals, need to participate in optimizing the impact of internet use on QoL.

First, since LIU can increase individuals’ QoL, the online entertainment industries and enterprises need to be regulated and supported. In particular, the innovation and promotion of high-quality online entertainment products are encouraged. Second, due to the side effects of LIU (e.g., risk perception and internet addiction), it is necessary for the government to regulate online entertainment enterprises and products to a certain extent [69], and provide the official guide to proper use of the Internet for the public. The government particularly needs to pay special attention to people with physical and mental health issues and severe internet addiction, and offer them counselling support and interventions to cope with internet addiction behaviors (e.g., constant online gaming and gambling) [70,71]. In the meantime, the government should also restrict excessively distorted internet information to avoid risk amplification among netizens. Frequent public education and training on healthy internet use should be provided by governments, educational institutions, social organizations, and communities to increase individuals’ abilities to filter false information. In addition, specific efforts need to be paid to the post-COVID-19 digital society. Quarantine policies and the reduction in physical interaction is likely to increase internet use and the risk of internet addiction [72]. The government or social organizations should provide specialized counselling services and psychological interventions for vulnerable groups so that they can perceive and judge the risks and respond sensibly [73]. Third, parents should take the accountability to set up ‘children/teenage mode’ for children to avoid too much exposure to risks on their physical and mental health [74].

## 6. Conclusions

Drawing on a psychological perspective and secondary survey data, we tested the complex influencing mechanisms of internet use on QoL through the two most characteristic factors of risk perception and internet addiction. Comparing the different influencing mechanisms of the two types of internet use (LIU/WIU) is largely absent from much of the existing research. Our findings reveal that different types of internet use had different impacts on QoL, while the relationships are affected by the serial mediating roles of risk perception and internet addiction. Our findings demonstrate important theoretical implications of researching the improvement of QoL in the digital society and post-COVID-19 era for human sustainability, as well as providing relevant and timely interventions for multiple stakeholders. In the future, the model needs to be tested with more samples from different areas, and a longitudinal study is necessary to enhance the credibility. Moreover, the boundary conditions of the relationship in the present model need to be discussed by exploring the moderating variables in the future studies.

## Figures and Tables

**Table 1:** Results of mean, SD, and ANOVA (*n* = 1535).

Socio-Demographics	LIU	WIU	RP	IA	QoL
All	5.33 (1.05)	4.06 (1.88)	3.24 (1.03)	2.59 (1.07)	3.58 (0.72)
Gender					
Men (*n* = 830)	5.27 (1.14)	4.06 (1.90)	3.11 (1.03)	2.54 (1.08)	3.57 (0.75)
Women (*n* = 705)	5.41 (0.93)	4.06 (1.84)	3.39 (1.00)	2.64 (1.06)	3.58 (0.67)
F	6.507 ^b^	0.000 ^a^	29.164 ^a^	3.303 ^a^	0.128 ^b^
P	0.011	0.996	<0.001	0.069	0.721
Age					
18–29 (*n* = 349)	5.57 (0.77)	4.64 (1.47)	3.16 (1.03)	3.10 (0.93)	3.71 (0.68)
30–39 (*n* = 335)	5.47 (0.98)	4.69 (1.60)	3.25 (1.02)	2.86 (1.04)	3.51 (0.72)
40–49 (*n* = 316)	5.27 (1.08)	4.19 (1.74)	3.31 (1.01)	2.53 (1.04)	3.51 (0.74)
50–59 (*n* = 299)	5.23 (1.18)	3.5 (2.01)	3.32 (1.02)	2.18 (1.01)	3.53 (0.74)
60 or older (*n* = 236)	5.02 (1.19)	2.84 (1.99)	3.16 (1.05)	2.04 (0.99)	3.62 (0.70)
F	13.210 ^b^	52.144 ^b^	1.851 ^a^	59.087 ^a^	4.985 ^a^
P	<0.001	<0.001	0.117	<0.001	0.001
Education					
<senior high school (*n* = 195)	5.08 (1.21)	2.42 (1.85)	3.52 (0.98)	2.07 (1.06)	3.52 (0.78)
senior high school (*n* = 448)	5.22 (1.11)	3.48 (1.86)	3.42 (1.06)	2.46 (1.10)	3.50 (0.78)
college (*n* = 359)	5.31 (1.10)	4.51 (1.64)	3.18 (0.99)	2.72 (1.06)	3.55 (0.68)
bachelor (*n* = 356)	5.53 (0.87)	4.69 (1.53)	3.04 (0.98)	2.81 (1.00)	3.69 (0.65)
master (*n* = 155)	5.55 (0.84)	5.18 (1.24)	3.03 (1.01)	2.86 (0.97)	3.66 (0.66)
>master (*n* = 22)	5.45 (0.80)	5.18 (1.37)	2.86 (0.92)	2.18 (0.68)	3.70 (0.57)
F	7.736 ^b^	79.576 ^b^	10.847 ^a^	18.888 ^b^	3.898 ^b^
P	<0.001	<0.001	<0.001	<0.001	0.002

Note. Standard deviations are in parentheses; LIU = leisure-oriented internet use; WIU = work-oriented internet use; QoL = quality of life; RP = risk perception; IA = internet addiction; ^a^. statistical analysis was performed using One-way ANOVA; ^b^. statistical analysis was performed using Welch test.

**Table 2:** Factor loading of items.

Construct	Item	Loading	CR	AVE
RP	RP1	0.791	0.887	0.662
	RP2	0.841		
	RP3	0.820		
	RP4	0.801		
IA	IA1	0.759	0.841	0.514
	IA2	0.740		
	IA3	0.742		
	IA4	0.720		
	IA5	0.616		
QoL	QoL1	0.842	0.842	0.641
	QoL2	0.840		
	QoL3	0.713		

Note. LIU and WIU were single-item constructs and were thus not included; CR = composite reliability; AVE = average variance extracted.

**Table 3:** Correlations for LIU, WIU, RP, IA, and QoL (*n* = 1535).

Variables	LIU	WIU	RP	IA	QoL
LIU	-				
WIU	0.338 **	-			
RP	0.056 *	−0.092 **	0.814		
IA	0.299 **	0.293 **	0.085 **	0.717	
QoL	0.057 *	0.022	−0.097 **	−0.059 *	0.784

Note. Diagonal elements are squared roots of AVE, * *p* <0.05, ** *p* < 0.01.

**Table 4:** Regression results on QoL, RP, and IA.

	Model 1 (QoL)		Model 2 (IA)		Model 3 (RP)	
	β	*p*	β	*p*	β	*p*
Gender	0.033	0.364	0.057	0.247	0.261 **	<0.001
Age	−0.022	0.133	−0.215 **	<0.001	−0.036	0.078
Education	0.052 **	0.002	0.028	0.207	−0.145 **	<0.001
LIU	0.056 **	0.003	0.201 **	<0.001	0.084 **	0.001
WIU	−0.015	0.210	0.072 **	<0.001	−0.034 *	0.037
RP	−0.058 **	0.001	0.097 **	<0.001		
IA	−0.067 **	<0.001				

Note. * *p* <0.05, ** *p* < 0.01.

**Table 5:** Bootstrap analysis of significance test on mediating effect (for LIU).

Path	Effect	Boot SE	CI = 95%		Significance
			LLCI	ULCI	
Direct effect	0.0500	0.0182	0.0143	0.0857	Significant
Indirect effect					
TOTAL	−0.0207	0.0057	−0.0324	−0.0099	Significant
path1: LIU- > RP- > QoL	−0.0039	0.0019	−0.0083	−0.0008	Significant
path2: LIU- > IA- > QoL	−0.0163	0.0054	−0.0276	−0.0064	Significant
path3: LIU- > RP- > IA- > QoL	−0.0004	0.0002	−0.0010	−0.0001	Significant

Note. Boot SE = bootstrap standard error, LLCI = lower limit confidence interval, ULCI = upper limit confidence interval.

**Table 6:** Bootstrap analysis of significance test on mediating effect (for WIU).

Path	Effect	Boot SE	CI = 95%		Significance
			LLCI	ULCI	
Direct effect	−0.0056	0.0112	−0.0276	0.0164	Not significant
Indirect effect					
TOTAL	−0.0049	0.0026	−0.0102	0.0001	Not significant
path1: WIU- > RP- > QoL	0.0010	0.0010	−0.0005	0.0038	Not significant
path2: WIU- > IA- > QoL	−0.0060	0.0024	−0.0112	−0.0015	Significant
path3: WIU- > RP- > IA- > QoL	0.0001	0.0001	<0.0001	0.0005	Significant

## Data Availability

Data was obtained from the 2019 “Taiwan Social Change Survey” (TSCS) with the theme of “Technology and Risk Society” hosted by the Academia Sinica’s Institute of Sociology.
